# Validation of the fear of COVID-19 scale in a central Balkan country - Serbia

**DOI:** 10.3389/fpubh.2022.972668

**Published:** 2022-08-23

**Authors:** Radica Zivkovic Zaric, Milan Zaric, Petar Canovic, Slobodan Jankovic, Milorad Stojadinovic, Nenad Zornic, Jelena Nesic, Marko Spasic, Dalibor Jovanovic, Martina Jug, Stefan Jakovljevic, Ana Pejcic

**Affiliations:** ^1^Faculty of Medical Sciences, University of Kragujevac, Kragujevac, Serbia; ^2^Clinical Center Kragujevac, Kragujevac, Serbia; ^3^Clinical Center Serbia, Belgrade, Serbia

**Keywords:** COVID-19, scale, fear, validation, anxiety

## Abstract

**Validation of the fear of introduction:**

High levels of fear of COVID-19 may be associated with increased levels of stress, anxiety, and depression, as well as decreased resilience and life expectancy.

**Objective:**

This study aimed to translate and confirm the Serbian version of the Fear of COVID-19 scale as well as to investigate its psychometric properties.

**Methods:**

The translation and intercultural adaptation of the Fear of COVID-19 scale was performed by the leading standard of the International Society for Pharmacoeconomics and Outcome Research. When the distribution was normal, the Kolmogorov-Smirnov test was used. The reliability of the Serbian version of FCV-19S was tested by measuring the internal consistency through the value of Cronbach's alpha.

**Results:**

The original version of the Fear of COVID-19 scale was tested on a sample of 256 subjects with a mean age of 25.38 ± 12.47. The Cronbach's alpha value was 0.864. We divided the scale by the split-half method (Spearman-Brown), and the value of the coefficient for the questionnaire as a whole was 0.882. Divergent criterion validity was tested through the non-parametric correlation between the scores of the Fear of COVID-19 scale and the Fear of Hospitalization scale. A score of the Fear of COVID-19 scale was calculated as the sum of each question for each of the respondents. Convergent criterion validity was tested through the non-parametric correlation between the scores of the Fear of COVID-19 scale and the Emotional Regulation Questionnaire.

**Conclusion:**

The validated version of the scale in Serbia complements versions available in other cultures and other languages and facilitates global studies related to mental health during the COVID-19 pandemic.

## Introduction

The new coronavirus disease (COVID-19) is a viral respiratory infection that was originally identified in Wuhan, China, in late 2019 (1). It quickly spread around the world and the World Health Organization officially declared a pandemic on March 11, 2020 (1). In Serbia, the first case of COVID-19 was registered on March 6, 2020, and as of January 22, 2021, the number of confirmed cases exceeded 380,800 with 3,849 deaths (2). The emergence of COVID-19 and its aftermath has led to concern and fear among individuals around the world (3). For potentially life-threatening situations, fear is a standard response that affects an individual's behavior in the face of a threat, but this response may be inadequate if the level of fear is extreme (4, 5). A high level of fear of COVID-19 can be related to enlarged levels of stress, anxiety, and depression, as well as decreased resilience and life satisfaction (4, 6, 7).

Assessment tools aimed at evaluating an individual's fear of COVID-19 have recently begun to emerge (8). Ahorsu et al. (3) developed a concise tool to specifically address the fear of COVID-19, the Fear of COVID-19 Scale (FCV-19S), which has rapidly become a widely used tool to assess the fear of COVID-19 (7). The FCV-19S offers high potential for international comparative research on the psychosocial responses to COVID-19 as it has already been translated to many languages and shown good psychometric characteristics, validity and reliability in various studies conducted worldwide [e.g. in Norway (9), Israel (8), China (10), Romania (11), Eastern Europe (12), Brazil (13), Argentina (4), Saudi Arabia (14), Japan (15), Greece (16), Ethiopia (17), Italy (18), Spain (19), Turkey (20), Malaysia (21), United States (22), Bangladesh (23), Peru (24), Pakistan (25), New Zealand (26), etc.]. Most of these investigations have suggested that this scale has a one-dimensional constitution, but some of the authors indicated the presence of two factors: the initial related to physiological responses to COVID-19 and the next that represents emotional responses to COVID-19 (4, 8, 12, 24, 27). With only seven items, the FCV-19S has a significant benefit of brevity and may be particularly useful in a demanding clinical setting because it can be quickly administered (27).

To investigate the aspects of fear of COVID-19 in the Serbian population, the translation and validation of the FCV-19S are particularly important. Therefore, this study aimed to translate and validate the Serbian version of the FCV-19S and to further explore its psychometric properties.

## Methods

All participants fulfilled information consent before entering the study. This research was approved by the Ethics Committee of the Faculty of Medical Sciences, the University of Kragujevac, Serbia (No 01-11061) and performed according to principles cited in the Declaration of Helsinki about experimentations on human subjects.

The final Serbian adaptation of FCV-19S was tested on students and employees of the Faculty of Medical Sciences, University of Kragujevac as well as on randomly selected people from the authors' environment. The study was performed from October 2020 until December 2020. Inclusion criteria were educated adults and exclusion criteria were: pregnancy, lactation, COVID-19 infection as well as developmental delay. Investigators gave questionnaires to the respondents to fill them by themselves on the spot.

Translation and cross-cultural adaptation of the FCV-19S were performed according to the guiding principle of the International Society for Pharmacoeconomics and Outcome Research (ISPOR) (28). We got permission for translation and cross-cultural adaptation from the authors of the Fear of COVID-19 scale (3). We also got permission for using the Fear of Hospitalization scale as well as for the Emotional Regulation Questionnaire (ERQ) (29, 30). The original version of the scale was translated from English into Serbian by two English speakers, a professional translator and a bilingual university professor. Especially from each other, they translated the questionnaire, and then they met and the translation was synchronized to one Serbian version. This Serbian version of the scale was then translated into English by English speakers. The researchers then compared these two versions of the questionnaire, the original and the Serbian, and the translation was accepted. The final version in Serbian was then tested on 10 respondents, and after minor changes, it was copied and distributed for testing. We also collected demographic data on respondents with a specially designed questionnaire.

Kolmogorov-Smirnov test was used if the answers were distributed normally, in contrary non-parametric tests were used. The reliability of the Serbian version of the FCV-19S was checked by measuring the inside solidity through the value of Cronbach's alpha. Convergent and divergent validity was assessed with the total score of the Fear of Hospitalization scale and ERQ. Method of principal axis factoring was used when answers were not normally dispersed in the Serbian translation of the Fear of COVID19 scale. According to the fact that eigenvalues had to be superior to 1, we extracted new factors and gave them names (31, 32).

The sample size was determined based on the desired study strength of 80%, the maximum probability of error of the first type of 0.05, and the use of Bonett's formula. The minimum required sample size is 167 subjects at Cronbach's alpha 0.82. The study of Italian authors was used to calculate the strength of the study (18).

Calculations in the research were performed by SPSS software, version 18, and the stage of importance is set to *p* < 0.05.

## Results

The original version of the Fear of COVID 19 scale was tested on the sample of 256 respondents with mean age 25.381 ± 2.47. Most of the respondents were females in 75.8% (*n* = 194) and unmarried in 75.3% (*n* = 183). Other demographic data are given in Table 1. Translated version of Fear of COVID 19 scale is visible in Table 2.

**Table 1:** Demographic characteristics of the subjects.

**Variable**	**Mean ±standard**
	**deviation (*n* = 256)**
Age (years)	25.381 ± 2.47
**Gender**	
Male	24.2% (*n* = 62)
Female	75.8% (*n* = 194)
**Marital status**	
Unmarried	75.3% (*n* = 183)
Married	21% (*n* = 51)
Divorced	3.3% (*n* = 8)
Widow	0.4% (*n* = 1)
**Education level**	
High school	79% (*n* = 201)
College	19% (*n* = 48)
PhD degree	2% (*n* = 5)
Employed	27.7% (*n* = 71)
Presence of chronic disease	12.2% (*n* = 19)
Contact with an infected	22.7% (*n* = 35)
person from COVID 19	

**Table 2:** Version of fear of COVID 19 scale on English and Serbian.

1. I am most afraid of coronavirus-19	1. Najvise se plasim koronavirusa-19
2. It makes me uncomfortable to think about coronavirus-19	2. Neprijatna mi je pomisao na koronavirus-19
3. My hands become clammy when I think about coronavirus-19	3. Znoje mi se dlanovi kada mislim o koronavirusu-19
4. I am afraid of losing my life because of coronavirus-19	4. Plasim se da cu izgubiti zivot zbog koronavirusa-19
5. When watching news and stories about coronavirus-19 on social	5. Kada gledam vesti i price na drustvenim mrezama o koronavirusu-19
media, I become nervous or anxious	postajem nervozan/na ili uznemirena/a
6. I cannot sleep because I'm worrying about getting coronavirus-19	6. Ne mogu da spavam jer brinem da cu dobiti koronavirus-19
7. My heart races or palpitates when I think about getting coronavirus-19	7. Moje srce brze radi ili preskace kada mislim da cu dobiti koronaviurs-19

### Completing the questionnaires

The usual time which respondents needed to fulfill the all questionnaires was about 15 min, and the duration of completing the Fear of COVID 19 scale was about 5 min. All of the 256 respondents answered to all the questions in all three questionnaires.

### Reliability

We tested the Fear of COVID 19 scale and we got results about mean values, variance, skewness and kurtosis about each of the questions (Table 3). The value of Cronbach alpha was 0.864. We separated the scale by split-half method (Spearman-Brown), and the value of the coefficient for the questionnaire as a whole was 0.882.

**Table 3:** Fear of COVID-19 scale; mean values, standard deviation, skewness and kurtosis of responses to items.

	**Number of patients**	**Mean**	**Std. deviation**	**Skewness**	**Kurtosis**
I am most afraid of coronavirus-19	256	2.51	1.11	0.34	−0.47
It makes me uncomfortable to think about coronavirus-19	256	2.78	1.26	0.93	−1.17
My hands become clammy when I think about coronavirus-19	256	1.70	0.87	1.52	2.83
I am afraid of losing my life because of coronavirus-19	256	1.98	0.99	0.94	0.59
When watching news and stories about coronavirus-19 on social	256	2.78	1.23	0.00	−1.06
media, I become nervous or anxious					
I cannot sleep because I'm worrying about getting coronavirus-19	256	1.47	0.73	2.34	7.84
My heart races or palpitates when I think about getting coronavirus-19	256	1.89	0.98	1.14	1.02

### Factor analysis

The value of Kaiser–Meyer–Olkin test (a measure of sampling adequacy) was 0.862 and Bartlett's test of sphericity was important (*p* < 0.001). With orthogonal rotation, two factors were extracted, showing in total 71.7% of the variance. The eigenvalue of the first factor was 3.99 (57.09 % of variance) and the eigenvalue of the second factor was 1.03 (14.07% of variance). The rotated part matrix is exposed in Table 4. When the factor was loading superior to one it represents the cut-off point for transmission. Items 3, 4, 6, 7 belong to the first factor and reflect physical signs of fear and items 1, 2, 5 belong to the second factor and indicate psychological signs of fear.

**Table 4:** The rotated component matrix of the Fear of COVID-19 scale with factor loading per each item.

	**Factors**	
	**Physical signs of fear**	**Psychological signs of fear**
I am most afraid of coronavirus-19	0.139	**0.648**
It makes me uncomfortable to think about coronavirus-19	−0.033	**0.743**
My hands become clammy when I think about coronavirus-19	**0.776**	−0.037
I am afraid of losing my life because of coronavirus-19	**0.552**	0.312
When watching news and stories about coronavirus-19 on social media, I become nervous or anxious	−0.024	**0.806**
I cannot sleep because I'm worrying about getting coronavirus-19	**0.910**	−0.092
My heart races or palpitates when I think about getting coronavirus-19	**0.644**	0.257

Bolded numbers within the cells belong to the corresponding factor.

### Validity

The non-parametric correlation was used according to the non-normal distribution of some of the scores. Divergent criterion validity was tested through the non-parametric correlation between the scores of the Fear of COVID-19 scale and the Fear of Hospitalization scale. A score of the Fear of COVID-19 scale was calculated as the sum of each question for each of the respondents. Convergent criterion validity was tested through the non-parametric correlation between the scores of the Fear of COVID-19 scale and the ERQ. Spearman's correlation coefficients are presented in the multi-trait, multi-method matrix (Table 5).

**Table 5:** Non-parametric Spearman's coefficients.

	**Fear of COVID-19 scale**	**ERQ**	**Fear of hospitalization scale**
Fear of COVID-19 scale	1	Rho = −0.040 *p* = 0.519	Rho = 0.402 *p* = 0.000
ERQ	Rho = −0.040 *p* = 0.519	1	Rho = −0.029 *p* = 0.646
Fear of hospitalization scale	Rho = 0.402 *p* = 0.000	Rho = −0.029 *p* = 0.646	1

## Discussion

In this study, we represent data from translation and validation of a Fear of COVID-19 scale to Serbian language and culture. It is the first time that this scale is translated and validated in some Balkan countries. This is very important because now, at this moment epidemic or better pandemic of COVID-19 is a burning issue in the world's population. Moreover, our results show that the Serbian translation of the Fear of COVID-19 scale is cross-culturally very suitable in Serbian speech areas.

Most of the participants in our study were females (75.8%) and the mean age of the respondents was 25.381 ± 2.47 years. Also, most of the participants were unmarried (75.3%) with a high school education (79%). According to the factor analysis, we revealed two factors (Physical signs of fear as well as Psychological signs of fear).

The Serbian translation of the Fear of COVID-19 scale showed outstanding reliability and inner stability with Cronbach's alpha of 0.864. Therefore, the internal consistency of the Serbian translation of the Fear of COVID-19 scale was very similar to other studies such as the Italian version (α = 0.87) (18), the Hebrew version (α = 0.86) (8), Bangla version (α = 0.87) (23) and Spanish version (α = 0.86) (33). Moreover, Cronbach's alpha of our study was higher in comparison to the Russian version (α = 0.80) (12) and the Persian version (α = 0.82) (3).

The results of the exploratory factor analysis exposed a two-factor construction model, and two factors explained 71.7% of the total variance. These factors integrated items representing Physical signs of fear as well as Psychological signs of fear. Even though earlier period analyses of this scale indicate a one-dimensional model (3, 23), our result gives support for a two-factor structure model, separating physical signs of fear from psychological signs of fear. Jointly, these two factors explained a superior proportion of the variance, as well as the study in Israel (8). This division may allow researchers and clinicians to distinguish between fear and the symptoms it causes. Such a distinction is important because it has been shown that fear symptoms can later lead to depression, anxiety, and abuse of various substances and alcohol (8).

The results of our study showed that the Serbian translation of the Fear of COVID-19 scale has suitable psychometric and statistical properties and is therefore, appropriate to be used for large-scale epidemiological and experimental design studies for psychological interventions in the Serbian population.

However, our study has several limitations. The first limitation is that the results were obtained by self-evaluation of the respondents, which may carry the risk of bias. Another limitation is that it is a cross-sectional study, so we do not have data on the history of fear over time. Also, the data were collected from persons who were not hospitalized, so the results of the study may not be generalized to hospitalized patients.

Regardless of its limitations, the results of this study should help health professionals cope with the mental health problems of public health affected by the COVID-19 pandemic. A recent study by Harper et al. (34) showed that increased fear of COVID-19 was associated with reduced quality of life and physical distancing as well as increased hand hygiene, suggesting that fear plays a significant role in encouraging people to adhere to COVID-19 public health recommendations. Moreover, public health interventions can be considered to mitigate the traumatic effects of COVID-19 by using the COVID-19 fear scale. Understanding the long-term relationship between aspects of the pandemic, both medical and social, and the resulting levels of anxiety would be a fruitful and important course of research.

In conclusion, the validated version of the scale in Serbia complements versions available in other cultures and other languages, and facilitates global studies related to mental health during the COVID-19 pandemic.

## Data availability statement

The raw data supporting the conclusions of this article will be made available by the authors, without undue reservation.

## Ethics statement

The studies involving human participants were reviewed and approved by Ethics Committee of the Faculty of Medical Sciences, the University of Kragujevac, Serbia (No 01-11061). The patients/participants provided their written informed consent to participate in this study.

## Author contributions

RZ: idea. MZ, SJ, NZ, JN, DJ, and AP: writing. MSt, PC, MSp, MJ, and SJ: collecting data. All authors contributed to the article and approved the submitted version.

## Conflict of interest

The authors declare that the research was conducted in the absence of any commercial or financial relationships that could be construed as a potential conflict of interest.

## Publisher's note

All claims expressed in this article are solely those of the authors and do not necessarily represent those of their affiliated organizations, or those of the publisher, the editors and the reviewers. Any product that may be evaluated in this article, or claim that may be made by its manufacturer, is not guaranteed or endorsed by the publisher.
